# Does Intraoperative Extracochlear Electrocochleography Correlate With Postoperative Audiometric Hearing Thresholds in Cochlear Implant Surgery? A Retrospective Analysis of Cochlear Monitoring

**DOI:** 10.1177/23312165241252240

**Published:** 2024-05-07

**Authors:** Sabine Haumann, Marlene Mynarek (née Bradler), Hannes Maier, Victor Helmstaedter, Andreas Büchner, Thomas Lenarz, Magnus J. Teschner

**Affiliations:** 1 Department of Otorhinolaryngology, 9177 Hannover Medical School, Hannover, Germany; 2 Cluster of Excellence “Hearing4All”, Hannover, Germany; 3 Department of Otorhinolaryngology, Proselis Klinikum Recklinghausen, Recklinghausen, Germany

**Keywords:** cochlear implant, hearing preservation, electrocochleography

## Abstract

In recent years, tools for early detection of irreversible trauma to the basilar membrane during hearing preservation cochlear implant (CI) surgery were established in several clinics. A link with the degree of postoperative hearing preservation in patients was investigated, but patient populations were usually small. Therefore, this study's aim was to analyze data from intraoperative extracochlear electrocochleography (ECochG) recordings for a larger group.

During hearing preservation CI surgery, extracochlear recordings were made before, during, and after CI electrode insertion using a cotton wick electrode placed at the promontory. Before and after insertion, amplitudes and stimulus response thresholds were recorded at 250, 500, and 1000 Hz. During insertion, response amplitudes were recorded at one frequency and one stimulus level. Data from 121 patient ears were analyzed.

The key benefit of extracochlear recordings is that they can be performed before, during, and after CI electrode insertion. However, extracochlear ECochG threshold changes before and after CI insertion were relatively small and did not independently correlate well with hearing preservation, although at 250 Hz they added some significant information. Some tendencies—although no significant relationships—were detected between amplitude behavior and hearing preservation. Rising amplitudes seem favorable and falling amplitudes disadvantageous, but constant amplitudes do not appear to allow stringent predictions.

Extracochlear ECochG measurements seem to only partially realize expected benefits. The questions now are: do gains justify the effort, and do other procedures or possible combinations lead to greater benefits for patients?

## Introduction

Minimizing insertion trauma to the cochlea, and achieving optimum intracochlear electrode positioning during cochlear implantation, are essential for preserving residual hearing and improving postoperative hearing outcomes (James et al., 2005). Over the years, advances in technology and optimized surgical procedures have led to better performance, in turn resulting in the expansion of selection criteria for cochlear implantation (Lenarz, Büchner et al., 2022; Wilson & Dorman, 2008). One avenue pursued was the development of atraumatic electrodes to preserve existing residual hearing, thus enabling the patient to have a combination of acoustic and electrical hearing (Adunka et al., 2004; Lenarz et al., 2020; Risi, 2018). This was accompanied by improvement in surgical techniques (Giordano et al., 2014; Khater & El-Anwar, 2017; Lehnhardt, 1993; Sikka et al., 2017). Another trend is the move away from cochleostomies unless they are unavoidable (e.g., due to ossification), because the approach through the round window is more favorable in terms of preserving residual hearing (Avasarala et al., 2022). Hence, there are increasing numbers of patients with significant residual hearing, particularly at the lower frequencies up to 1.5–2 kHz (Briggs et al., 2006; Lenarz, Büchner et al., 2022; Lenarz et al., 2009). In this patient group, hearing preservation has become of the utmost importance in terms of improved speech understanding, especially in noise, and potentially improving music appreciation (possibly due to perception of low-frequency speech formants and lower harmonic frequencies in music) (Braun et al., 2011; DeMason et al., 2012; Gstoettner et al., 2008). However, there is a potential risk of losing residual hearing immediately during or after surgery (due to insertion trauma) or later (as a result of delayed inflammatory or foreign-body reactions; Braun et al., 2011).

For these reasons, it would be beneficial to alert the surgeon, at a sufficiently early stage, of a risk of trauma, as this often does not become apparent until after damage has already occurred. One possible approach is to use electrocochleography (ECochG) with acoustic stimulation (Barnes et al., 2021; Choudhury et al., 2012). This form of ECochG is already known from preoperative diagnostics, in which context it allows single recordings of the cochlear microphonics (CMs), summating potentials (SPs), compound action potentials (CAPs), and auditory nerve neurophonics (ANN). Here, CMs are known to be primarily outer hair cell responses and represent current flow through the mechanoelectric transducer channels in the stereocilia (Patuzzi et al., 1989; Verpy et al., 2008). SPs are generally considered to be an inner hair cell response at low intensities and a mixed response from inner and outer hair cells at high intensities and represent sustained depolarization in the hair cell body during sound presentation (Durrant et al., 1998; Zheng et al., 1997). The CAP, derived from auditory nerve fibers, constitutes the sum of synchronous responses to the onsets and offsets of sounds (Choudhury et al., 2012; Eggermont, 1976; Schoonhoven et al., 1995). Earlier studies suggested that CAPs, in particular, serve as early markers of electrode interaction with cochlear structures in gerbils (Adunka et al., 2010; Helmstaedter et al., 2018). Unfortunately, CAPs can be recorded more reliably in the higher-frequency range, whereas the residual hearing of many CI patients lies in the low-frequency range. A study aiming to assess CAPs in human CI subjects was able to detect measurable CAPs at any frequency in only about half of participants; even where present, they were highly variable (Scott et al., 2016). ANNs are a phase-locked response to auditory stimuli and hence regarded as reflecting the status of the auditory nerve fibers, they occur especially in the low-frequency range. CMs and ANNs are difficult to distinguish and are often referred to jointly as ongoing responses (ORs). Thus, ORs are the most commonly measured ECochG component with respect to hearing and speech outcome (Dalbert et al., 2021; Giardina et al., 2018; Harris et al., 2017; Haumann et al., 2019; Scott et al., 2016; Walia et al., 2022).

A limitation of all intraoperative recordings is the fact that CI insertion can elicit postoperative inflammatory processes (Seyyedi & Nadol, 2014; Simoni et al., 2020). It is therefore still possible that, where a stable ECochG signal is detected, the residual cochlear function is preserved at the end of the surgery, but deteriorates afterward. In a recent study, the bone conduction threshold was measured within 5 hours of surgery being completed (Saoji et al., 2022). This early measurement enabled the effect to be investigated further.

Stimulation is usually carried out acoustically via an insert earphone placed in the external auditory canal using tone bursts of different frequencies and clicks. There are two primary approaches adopted in the recording. In one approach, recording takes place extracochlearly: this involves a recording electrode being placed at or close to the promontory wall and, usually, being connected to a clinical auditory brainstem response (ABR) device for stimulating and recording (Adunka et al., 2006; Choudhury et al., 2012; Dalbert et al., 2018; Dalbert et al., 2020; Fitzpatrick et al., 2014; Giardina et al., 2018; Harris, Cruise, Gibson, Bate & Sanli, 2011; Haumann et al., 2019; Mandalà, Colletti, Tonoli & Colletti, 2012; Suntinger et al., 2022). This electrode stays in place throughout CI electrode insertion. This is also the main advantage of the approach. If the recorded signal changes, it can be assumed that there is something abnormal going on with the electrode insertion. Sudden amplitude reductions have been interpreted as physiological markers of electrode contact with cochlear structures in gerbils (DeMason et al., 2012). Studies in human subjects have also been performed which concluded that reductions in electrocochleographic magnitude during cochlear implantation might indicate cochlear trauma (Calloway et al., 2014; Dalbert, Huber, Veraguth, Roosli & Pfiffner, 2016); this could, however, also be due to the CI electrode temporarily “blocking” the basilar membrane for example, by mechanically dampening it during the ongoing insertion process. Although several research groups have investigated this method, most of them stated that a large trauma is likely to be detected, but also that a preserved ECochG signal does not necessarily mean that the residual hearing of the patient was also preserved (Adunka et al., 2016; Dalbert et al., 2015; Radeloff et al., 2012). However, few other studies have detected significant correlations to residual hearing (Dalbert et al., 2018; Fitzpatrick et al., 2014) or speech perception (Abbas, Tejani, Scheperle & Brown, 2017; Walia et al., 2022). Most groups looked at amplitudes and thresholds of the extracochlearly recorded signal, but one group investigated the phase of the signal: here, correlations were detected between phase changes and hearing preservation, with changes in phase being correlated with worse outcome (Suntinger et al., 2022). Practical disadvantages of extracochlear recordings include the challenge of securing the recording electrode in place such that it genuinely does not move during surgery. Another difficulty relates to the precise location of the recording site. The best signals can be recorded from the round window but, as the CI electrode is usually inserted through the round window, the electrode cannot remain in place during insertion (Giardina et al., 2018; Walia et al., 2022). When the electrode was placed too close to the stapes at the oval window, the oscillations of the incoming sound waves also led to movement of the recording electrode and, in turn, to oscillation of the impedance between the recording electrode and recording site. Thus, an artifact that looks very similar to the recorded response signal is induced, especially at stimulation levels from 80 dB nHL upward, such that the two are virtually indistinguishable (Teschner, Lenarz & Battmer, 2012). Attempts have been made to remedy this by using cotton wick electrodes, in which the tip of the metal electrode is wrapped in wet medical cotton (Calloway et al., 2014; Haumann et al., 2019; Mandalà, Colletti, Tonoli & Colletti, 2012). While this reduces the risk of false positive responses, it does not eliminate it. Another relevant disadvantage of extracochlear recordings is that, at least in part, the measurement set-up has to be developed in-house, limiting the ease of operation.

There has, therefore, been an increasing trend toward adoption of a second, different approach using intracochlear recordings (Buechner et al., 2022; Calloway et al., 2014; Campbell, Kaicer, Briggs & O’Leary, 2015; Dalbert et al., 2018; Harris et al., 2017; Haumann et al., 2019; Haumann, Timm, Büchner, Lenarz & Salcher, 2024; Koka, Saoji & Litvak, 2017; Lenarz et al., 2022; Saoji et al., 2022). Here, recording takes place much closer to the relevant structures in the cochlea, yielding substantially larger amplitudes. Also relevant is the fact that the cochlea is fluid filled. Thus, even if the recording electrode oscillates due to exposure to the traveling wave, this does not lead to stimulus-synchronous oscillations in impedance, hence preventing artifacts. The main disadvantage of this approach is that the recording site moves during CI electrode insertion. Thus, it is currently not known how the recorded signal should behave for optimal hearing preservation, and there are no known patterns of changes between recorded amplitudes at the beginning and end of CI electrode insertion that reliably predict outcomes. In theory, the recorded amplitude should increase as ongoing electrode insertion proceeds as the intracochlear recording electrode approaches the tonotopic region of the low-frequency stimuli. However, any drop in amplitude could also be caused by changes relating to the recording site, such as when the characteristic frequency (CF) of the stimulus in the cochlea and therewith the main signal generator of the response is exceeded by the recording electrode. In recent years, CI companies have developed integrated solutions whereby intracochlear recording involves telemetric control of the clinical CI electrode using the clinical stimulation coil, as well as convenient hardware and software solutions.

Nevertheless, the advantage of extracochlear recording—namely, that the recording site does not move—means this method will remain the subject of further investigation. Drawing on heterogenous findings with respect to the usefulness of extracochlearly recorded ECochG, the present work attempts to correlate such recordings with postoperative audiometric hearing thresholds. It does so using a promontory wall electrode for extracochlear recordings throughout the entire surgical process of cochlear electrode insertion, and on a patient group large enough to be statistically meaningful.

## Materials and Methods

The routine clinical procedure involves recording acoustically evoked responses from the promontory using a cotton wick electrode during cochlear implantation. This retrospective analysis includes 121 data sets from adult patients with residual hearing who underwent regular cochlear implantation. Patients with irregular temporal bone anatomy were excluded from the analysis. The study was approved by the local ethics committee (approval number 1897-2013) and is in accordance with the ethical standards of the Declaration of Helsinki.

### Required Sample Size

A power analysis was used to determine how large the sample needs to be to allow statistical analysis that can statistically obtain an outcome with the expected effect size. The G*Power program was used to calculate the necessary sample size (Faul, Erdfelder, Buchner & Lang, 2009). Spearman's rank correlation is computationally identical to Pearson's product-moment correlation, so the power analysis was performed on the product-moment correlation.

The analysis determined how large the sample must be depending on the expected level of correlation to achieve a power of β = 0.90, that is, the a priori probability of discovering an existing connection is 90%.

Correlation coefficients from an earlier study (Haumann et al., 2019) were used to estimate the expected correlation. These coefficients were taken from Table 5 in Haumann et al. (2019), specifically the first line (preoperative audiogram vs. pre-insertion EC (extracochlear) OR), fourth line (postoperative audiogram at test tone vs. post-insertion EC OR), and fifth line (postoperative audiogram at test switch-on vs. post-insertion EC OR). The correlation coefficients for all three frequencies ranged from *r* = .05 to *r* = .76, and the mean of all nine entries was *r* = .3, which was used for the current analysis. Power analysis showed that, with an expected mean correlation of ρ = 0.3, a sample size of *N* = 112 is necessary.

In the present work, the intention was to ascertain these mean relationships (ρ ≥ 0.30) between extracochlear electrocochleography and tone audiometry, so that the sample size had to be at least *N* = 112. (This was achieved, the actual sample size being *N* = 121.)

### Patients

Of the 114 patients included, 55 (48.2%) were men and 59 (51.8%) women. Seven received a bilateral implantation, so the total number of ears implanted was 121. The mean age at the time of implantation was 50.7 years (median 56.0, standard deviation ± 21.73). Implants from all four manufacturers approved in Germany were implanted. Both the electrode and the manufacturer were selected taking into account individual physiological and anatomical conditions, as well as the patient's wishes. Of the patients, 30.6% received an implant from cochlear, 44.6% from MED-EL, 19.8% from Advanced Bionics, and 5.0% from Oticon Medical/Neurelec SAS. Detailed data on the patients is given in Table 1.

**Table 1.** Demographic, Audiometric, and Electrophysiological Data.

Subject (no.)	Sex (female/male)	Age at surgery (years)	Side (left/right)	Implant type	Pre-surgical PTA low (dB HL)	TSO PTA low (dB HL)	FF PTA low (dB HL)	ECochG Threshold shift low (dB nHL)	ECochG stim freq dur ins (Hz)	ECochG stim level dur ins (dB nHL)	ECochG Amplitude shift (µV)
1	m	56	L	Synchrony Flex 28	61.67	91.67	95.00	only pre	1000	100	0.36
2	f	53	L	HiRes Ultra SlimJ	60.00	91.67	110.00	−6.33	n/a	n/a	n/a
3	m	1	R	Nucleus CI 522	n/a	n/a	n/a	−10	500	70	−0.29
4	m	1	L	Nucleus CI 522	n/a	n/a	n/a	7	500	70	−0.34
5	f	83	R	Synchrony Flex 28	61.67	96.67	n/a	nr	n/a	n/a	n/a
6	m	9	L	Synchrony Flex 16	23.33	n/a	61.67	−3.36	500	90	0.02
7	m	70	L	HiRes Ultra Mid-Scala	68.33	93.33	103.33	2	n/a	n/a	n/a
8	f	39	R	HiRes 90 K Advantage Mid-Scala	43.33	93.33	116.67	0	n/a	n/a	n/a
9	f	38	L	Synchrony Flex 28	51.67	60.00	85.00	6.7	n/a	n/a	n/a
10	f	58	R	Neuro ZTI EVO	50.00	96.67	110.00	−5	500	86	0.07
11	f	26	R	Nucleus CI 522	35.00	36.67	98.33	22.66	1000	95	−0.06
12	m	56	L	Synchrony Flex 28	80.00	116.67	116.67	3.33	500	90	0.17
13	f	54	L	Nucleus CI 522	75.00	103.33	n/a	4	n/a	n/a	n/a
14	f	67	R	Nucleus CI24RE Hybrid-L	58.33	98.33	90.00	33.33	500	90	0.19
15	f	77	R	Synchrony Flex 24	58.33	105.00	90.00	−3.33	1000	96	0.02
16	m	17	L	Nucleus CI 522	75.00	110.00	n/a	−2	1000	95	0.00
17	f	66	L	HiRes 90 K Advantage Mid-Scala	83.33	116.67	93.33	5	1000	100	0.25
18	m	50	L	Neuro ZTI EVO	73.33	95.00	96.67	−9.66	1000	102	0.39
19	m	6	L	Nucleus CI 522	61.67	n/a	31.67	0	1000	70	0.64
20	m	6	R	Nucleus CI 522	46.67	n/a	58.33	4	1000	70	0.36
21	f	33	L	Nucleus CI 522	55.00	90.00	71.67	−6.67	1000	70	0.96
22	m	63	L	Synchrony Flex 24	48.33	81.67	76.67	nr	n/a	n/a	n/a
23	m	70	R	Nucleus CI 522	76.67	101.67	116.67	nr	n/a	n/a	n/a
24	m	53	R	HiRes Ultra SlimJ	66.67	83.33	73.33	−16	n/a	n/a	n/a
25	f	39	R	HiRes Ultra SlimJ	38.33	73.33	45.00	4	n/a	n/a	n/a
26	f	41	L	HiRes Ultra SlimJ	53.33	90.00	76.67	0	n/a	n/a	n/a
27	m	26	R	Nucleus CI 522	63.33	116.67	91.67	nr	n/a	n/a	n/a
28	f	42	L	Nucleus CI 522	88.33	105.00	103.33	11.67	n/a	n/a	n/a
29	f	49	L	Nucleus CI 522	103.33	110.00	106.67	3.33	500	70	0.39
30	f	50	R	Nucleus CI 522	90.00	90.00	n/a	1.66	1000	70	0.08
31	f	65	R	Nucleus CI 522	76.67	116.67	n/a	8.33	n/a	n/a	n/a
32	f	25	L	Concerto Flex EAS 20	73.33	101.67	88.33	3	n/a	n/a	n/a
33	m	69	L	HiRes Ultra SlimJ	63.33	85.00	101.67	−5.33	n/a	n/a	n/a
34	f	69	L	HiRes Ultra SlimJ	46.67	78.33	80.00	nr	n/a	n/a	n/a
35	m	47	R	Synchrony Flex 24	43.33	73.33	65.00	−1	1000	86	0.09
36	m	70	L	Nucleus CI 522	71.67	103.33	n/a	nr	n/a	n/a	n/a
37	m	45	R	Nucleus CI 522	95.00	105.00	105.00	3.33	500	80	0.19
38	m	45	L	Nucleus CI 522	90.00	115.00	95.00	−1.67	500	90	−0.18
39	f	54	L	HiRes Ultra SlimJ	88.33	115.00	116.67	0	1000	90	−1.41
40	m	81	R	Neuro ZTI EVO	61.67	85.00	88.33	−9.67	1000	100	0.11
41	f	57	L	Nucleus CI 522	80.00	105.00	83.33	1.67	1000	90	0.16
42	f	57	R	Nucleus CI 532	61.67	85.00	75.00	7	n/a	n/a	n/a
43	f	24	L	Synchrony Flex 16	36.67	70.00	116.67	10	1000	90	0.00
44	m	6	L	Nucleus CI 522	55.00	78.33	n/a	−3.33	1000	90	0.15
45	m	60	R	Synchrony Flex 24	50.00	73.33	81.67	−8	500	95	0.05
46	f	65	L	Synchrony Flex 24	93.33	116.67	116.67	−5	1000	100	0.25
47	m	3	R	Nucleus CI 522	90.00	n/a	n/a	0	1000	90	−0.06
48	f	66	L	Nucleus CI 522	60.00	86.67	68.33	−5	500	90	0.04
49	f	75	R	Nucleus CI 522	116.67	n/a	116.67	only pre	1000	105	0.09
50	f	35	R	HiRes Ultra SlimJ	70.00	101.67	91.67	13.33	n/a	n/a	n/a
51	f	60	L	Synchrony Flex 28	70.00	106.67	110.00	8.34	n/a	n/a	n/a
52	m	72	L	Synchrony Flex 24	45.00	96.67	55.00	0	500	90	0.38
53	f	59	L	Nucleus CI 532	61.67	93.33	83.33	nr	n/a	n/a	n/a
54	f	57	R	Nucleus CI 532	75.00	105.00	83.33	4.9	n/a	n/a	n/a
55	m	58	L	Synchrony Flex 28	51.67	98.33	68.33	−3	n/a	n/a	n/a
56	m	80	L	Synchrony Flex 24	55.00	90.00	116.67	3.3	500	95	0.03
57	f	67	L	Nucleus CI 522	103.33	110.00	n/a	nr	n/a	n/a	n/a
58	f	60	L	Synchrony Flex 28	75.00	111.67	98.33	15	500	80	0.57
59	m	68	R	Synchrony Flex 28	68.33	110.00	88.33	1.67	500	85	−0.06
60	m	56	L	Nucleus CI 522	78.33	98.33	88.33	−1.33	1000	97	0.14
61	m	77	L	Synchrony Flex 28	68.33	116.67	113.33	0	1000	100	−0.05
62	m	73	L	Synchrony Flex 24	60.00	83.33	n/a	9	500	70	0.40
63	f	19	R	HiRes Ultra SlimJ	88.33	96.67	93.33	0	n/a	n/a	n/a
64	m	60	R	Nucleus CI 522	63.33	76.67	61.67	−6.67	1000	80	0.01
65	f	91	L	Neuro ZTI EVO	71.67	111.67	96.67	3	1000	80	−0.13
66	f	52	R	Synchrony Flex 16	26.67	78.33	68.33	−5	n/a	n/a	n/a
67	m	63	R	Synchrony Flex 24	36.67	76.67	48.33	0	500	70	0.00
68	f	53	R	Nucleus CI 522	86.67	96.67	108.33	3.33	1000	89	0.08
69	m	0	R	HiRes Ultra SlimJ	n/a	n/a	n/a	1.33	n/a	n/a	n/a
70	f	49	L	Nucleus CI 522	48.33	86.67	68.33	−17	1000	96	−0.22
71	f	68	R	Nucleus CI 522	85.00	116.67	n/a	3.67	500	94	0.22
72	f	64	R	Neuro ZTI EVO	66.67	93.33	83.33	−1.34	1000	90	0.10
73	m	61	R	HiRes Ultra Mid-Scala	60.00	113.33	116.67	4.99	n/a	n/a	n/a
74	f	70	L	Nucleus CI 522	51.67	86.67	101.67	nr	n/a	n/a	n/a
75	m	77	L	Nucleus CI 522	48.33	116.67	108.33	16.33	500	85	−0.14
76	f	67	L	HiRes 90 K Advantage Mid-Scala	75.00	116.67	103.33	−8.33	n/a	n/a	n/a
77	f	51	L	HiRes Ultra SlimJ	90.00	116.67	116.67	0	n/a	n/a	n/a
78	m	53	R	Synchrony Flex 24	65.00	106.67	96.67	1.67	500	70	0.47
79	m	59	R	Nucleus CI 522	53.33	88.33	70.00	nr	n/a	n/a	n/a
80	m	68	L	HiRes Ultra Mid-Scala	116.67	n/a	n/a	0	n/a	n/a	n/a
81	m	72	R	Synchrony Flex 24	76.67	111.67	108.33	6.67	1000	100	0.21
82	f	30	L	Synchrony Flex 24	63.33	71.67	86.67	0	1000	100	−0.33
83	f	74	R	Synchrony Flex 24	55.00	80.00	106.67	30	n/a	n/a	n/a
84	m	8	R	Nucleus CI 522	55.00	81.67	81.67	0	500	70	0.39
85	m	58	R	Synchrony Flex 28	60.00	103.33	116.67	−3.3	500	90	−0.45
86	f	71	L	Synchrony Flex 28	76.67	95.00	n/a	12	n/a	n/a	n/a
87	f	37	L	Neuro ZTI EVO	83.33	116.67	116.67	6.34	500	85	−0.41
88	f	47	L	Nucleus CI 522	78.33	103.33	98.33	11.33	500	90	0.34
89	m	6	L	Synchrony Flex 24	40.00	65.00	n/a	14.67	1000	89	0.04
90	f	33	L	Synchrony Flex 20	58.33	88.33	65.00	2	1000	100	−0.04
91	m	68	L	HiRes Ultra SlimJ	45.00	70.00	53.33	n	n/a	n/a	n/a
92	f	51	L	HiRes Ultra SlimJ	90.00	108.33	n/a	−2	n/a	n/a	n/a
93	m	45	L	Synchrony Flex 28	95.00	108.33	116.67	−8	n/a	n/a	n/a
94	f	45	R	Synchrony Flex 24	43.33	70.00	61.67	16.7	500	70	0.38
95	m	22	R	HiRes Ultra SlimJ	86.67	103.33	98.33	−3.33	n/a	n/a	n/a
96	f	33	L	HiRes Ultra SlimJ	71.67	93.33	66.67	5	n/a	n/a	n/a
97	m	61	L	HiRes 90 K Advantage Mid-Scala	75.00	116.67	n/a	1.66	n/a	n/a	n/a
98	m	69	L	Synchrony Flex 24	55.00	63.33	65.00	−3.3	500	90	−0.33
99	f	41	L	Nucleus CI 522	60.00	101.67	n/a	−8.33	500	90	0.20
100	f	5	L	Synchrony Flex 24	86.67	91.67	n/a	0	1000	83	−0.35
101	f	12	R	Synchrony Flex 24	48.33	68.33	63.33	5	1000	70	0.23
102	m	87	R	Synchrony Flex 24	51.67	80.00	98.33	0	1000	70	−0.18
103	m	47	R	HiRes 90 K Advantage Mid-Scala	66.67	111.67	86.67	3	n/a	n/a	n/a
104	m	78	L	HiRes Ultra SlimJ	68.33	113.33	n/a	9	n/a	n/a	n/a
105	m	14	R	Synchrony Flex 24	40.00	103.33	100.00	0	1000	90	−0.09
106	m	72	R	Synchrony Flex 28	83.33	103.33	n/a	0	n/a	n/a	n/a
107	f	34	R	Synchrony Flex 28	86.00	108.33	105.00	−6.67	1000	90	0.11
108	f	76	L	Synchrony Flex 28	75.00	106.67	86.67	−5	500	90	0.07
109	f	71	R	Synchrony Flex 28	83.33	111.67	111.67	4.67	n/a	n/a	n/a
110	m	69	L	Synchrony Flex 24	55.00	63.00	65.00	−5	500	90	0.04
111	f	55	L	Synchrony Flex 28	53.00	73.00	78.00	0	n/a	n/a	n/a
112	f	43	R	Synchrony Flex 28	58.00	92.00	72.00	nr	n/a	n/a	n/a
113	m	47	R	Synchrony Flex 24	43.00	68.00	63.00	−5	n/a	n/a	n/a
114	m	68	R	Synchrony Flex 24	50.00	83.00	68.00	18.67	n/a	n/a	n/a
115	m	50	L	Synchrony Flex 28	68.33	116.67	105.00	10.34	500	96	−0.30
116	m	41	R	Synchrony Flex 28	72.00	102.00	106.67	15	1000	90	0.65
117	f	36	R	Synchrony Flex 28	98.33	116.67	98.33	3.33	1000	100	0.43
118	f	36	L	Synchrony Flex 28	93.33	116.67	101.67	0	1000	100	0.35
119	f	73	R	Synchrony Flex 28	70.00	116.67	116.67	16.3	1000	100	0.02
120	m	61	L	Synchrony Flex 28	58.33	108.33	116.67	4.5	n/a	n/a	n/a
121	f	56	L	Synchrony Flex 28	81.67	111.67	116.67	2.5	1000	100	−0.22

*Note:* TSO: test switch-on; FF: first fitting; only pre: ECochG threshold recording was carried out only preoperatively; nr: no responses even before insertion, so the remainder of the protocol was omitted; n/a: not applicable, recording was carried out intracochlearly, recording was omitted because of no responses, or the written protocol could not be evaluated because the markers for the specified insertion steps were missing.

### Pure-Tone Audiometry

The audiometric hearing threshold was determined, using pure-tone audiometry, at three time points: preoperatively (usually 1–2 days before implantation), at the time referred to as “test switch-on” (TSO) (i.e., on the first to third postoperative day), and at first fitting (FF) of the processor (usually 5 weeks after surgery). Hearing thresholds in air conduction at 250 Hz, 500 Hz, and 1000 Hz were used for analysis. The mean value across all three frequencies was calculated, for the above-mentioned measurement times, as pure-tone average (PTA low). Based on the differences between this mean value before and after surgery, or before surgery versus TSO, allocation to one of three categories of residual hearing preservation was made similar to that by Suhling et al. (2016). These groups were as follows: group 1: 0–15 dB drop in the low-frequency hearing threshold; group 2: ˃ 15–30 dB drop; and group 3: > 30 dB drop.

### Surgery and Recording Set-up for ECochG

**Figure 1.**
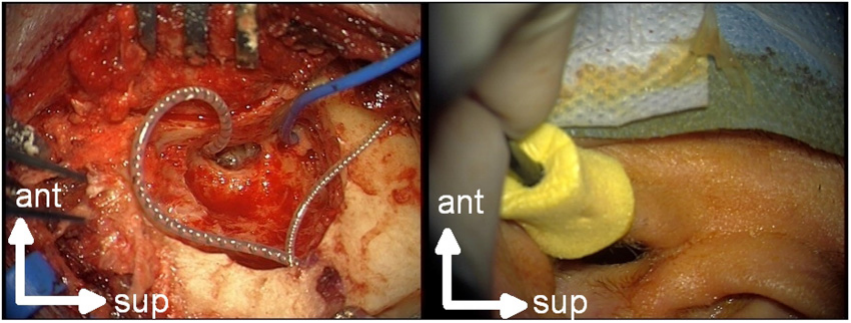
Extracochlear electrocochleography in situ. Left: view of the surgical site. The cable of the “cotton wick” measuring electrode is blue and the CI electrode is silver. Right: placement of the inserted phone in the ear canal. The arrows indicate the anterior (ant) and superior (sup) direction. Both panels show a left ear.

In accordance with our standard procedure, a transmastoid surgical approach with a posterior tympanostomy was used (Lenarz, 2018). The bone bed was created posterior–superior to the mastoid and the implant body was placed in the bone bed. A sterile foam insert transducer plug was placed in the external auditory canal just before CI electrode insertion. A sterile disposable steel wire (Medtronic, MI, USA) served as the recording electrode. Its end was de-insulated and twisted into a small piece of medical cotton, the aim being to increase the recording surface and reduce impedance fluctuations. The electrode was placed onto the promontory close to the round window via the antrotomy and was kept moist throughout the recording process by means of physiological saline (Figure 1). To reduce artifacts, contact between the recording electrode with the ossicular chain was avoided. The reference needle electrode was placed onto the vertex and the ground needle electrode onto the forehead.

The Nicolet Viking EDX system (Natus Medical Incorporated, Pleasanton, CA, USA) was used to generate acoustic stimuli and to record electrical potentials. Stimulation was performed using insert earphones (Nicolet TIP300, Natus Medical Incorporated, Pleasanton, CA, USA) placed in the external auditory canal. The stimuli were calibrated to normal hearing level (nHL). Typically, the series of frequencies was first tested at 70 dB nHL. When there were detectable responses, the stimulus intensity was gradually decreased by increments of 10 dB to visually determine the threshold at the tested frequency. The stimuli were tone bursts of different lengths, depending on frequency. Specific parameters and filters for the recording protocol are shown in Table 2. The repetition rate was 19.1 Hz and the recording window length 15 ms; the polarity was set to rarefaction and the number of averaged trials per trace was set to 10. The aim was to record CMs; however, especially in the low-frequency range, CMs and ANNs are difficult to distinguish, so overall we measured what are termed ongoing responses (ORs).

After the intraoperative situs had been prepared and the measurement set-up initiated, pre-insertion recordings were performed at different stimulation intensities across all frequencies. The aim here was to identify ORs within the complex electrocochleographic signal and to define their stimulus-response thresholds at specific frequencies. The stimulation frequency yielding the best results (distinct ORs with a low level of artifacts)—which proved to be 500 Hz or 1000 Hz—was chosen for the recordings performed during the CI electrode insertion process. The degree of stimulation was set to the minimum level that yielded distinct responses. Both individually chosen frequencies and loudness levels are given in Table 1. In all cases, the electrode was inserted through the round window. Recording during insertion was carried out throughout the entire insertion process but, to obtain comparable data, amplitudes at defined points in the process were analyzed: (1) before the round window was opened; (2) after the round window was opened; (3) when insertion of the lead commenced; (4) when the electrode was 25% inserted; (5) when the electrode was 50% inserted; (6) when the electrode was 75% inserted; (7) when the electrode was 100% inserted; (8) when the lead had reached its final position. After the electrode had been fully inserted, threshold recordings were repeated at all frequencies.

Following these measurements, the operation was completed as standard by placing the electrode into its final intramastoid position and by closing the wound in several layers. Implant integrity, electrode impedance, auditory nerve responses (using electrically evoked compound action potentials), and stapedius reflexes at selected electrode contacts were tested according to our standard operating procedures. Intraoperative cone-beam computed tomography (CBCT) of the temporal bone was performed to verify that the electrode was in the correct intracochlear position.

### Data Analysis

The raw data were exported from the recording system and analyzed with custom-written MATLAB routines; for details see Haumann et al. (2019). The frequency spectrum of each signal was obtained by mathematical analysis—specifically, by means of Fast Fourier Transform (FFT)—using MATLAB algorithms. The OR threshold was defined manually by an experienced audiologist using both the time-based plot and the frequency plot obtained by FFT. It was the lowest stimulation level at which distinct OR amplitudes were detectable. To analyze the amplitudes and compare them during the course of electrode insertion, typically the ORs observed 10–20 dB above the threshold were used.

To analyze the amplitude curve during insertion, a linear regression line was determined from the amplitudes recorded at the predefined substeps of the insertion (see above): (1) before the round window was opened; (2) after the round window was opened; (3) when insertion of the lead commenced; (4) when the electrode was 25% inserted; (5) when the electrode was 50% inserted; (6) when the electrode was 75% inserted; (7) when the electrode was 100% inserted; (8) when the lead had reached its final position. Four gradient types were defined based on the difference between the start and end point of the regression line. Where the difference exceeded 0.1 µV, the gradient type was defined as “rising.” If the difference was between −0.1 µV and 0.1 µV, the gradient type was described as “constant.” Where the difference was −0.1 µV or less, the gradient type was deemed to be “falling.” The fourth group was defined as a drop in amplitude with complete loss of signal. Where no stimulus response was observed at the highest level, the response was set to 10 dB above the stimulation limit (see Table 2). This approach was also used for pure-tone audiometry.

Statistical analysis was performed using SPSS version 26 (IBM, Ehningen, Germany). The significance level of the study was set at α = 0.05. For the ordinal variables of the individual hearing preservation groups, a cross-tabulation was created and the chi-square test was calculated. Means and standard deviations were calculated for all metric variables, that is, all hearing thresholds. To carry out further tests, a normal distribution analysis (applying Shapiro–Wilk's test and Kolmogoroff–Smirnoff's test), and a graphic assessment, were carried out. Since, according to this test, the metric variables were not subject to a normal distribution, the two-tailed Wilcoxon test was used to test for differences in position. This is particularly well suited for the comparison of two connected samples that are not necessarily normally distributed, as was the case in this study due to the different measurement times. In addition, to investigate a relationship between two variables, the Spearman correlation was calculated. The correlation results were classified after Cohen (1988): small effect: *r* = .1; mean effect: *r* = .3; large effect: *r* = .5.

Multivariate analyses were performed using a one-way analysis of variance (ANOVA) with preoperative audiometric thresholds, pre- and post-insertion OR thresholds and the OR threshold differences between post- and pre-insertion as input metrics, and the postoperative hearing preservation group as the output metric. First, the homogeneity of variance was verified using the Levene test. If homogeneity was confirmed (*p* > .05), the standard ANOVA was used and, where testing yielded a significant difference, post hoc analysis was carried out using Tukey's HSD test. If homogeneity was not confirmed (*p* < .05), Welch's ANOVA was used and, if the result yielded a significant difference, post hoc analysis was conducted by means of the Games-Howell test. This analysis was performed for all three frequencies individually as well as the mean, and it was carried out for the hearing preservation group at the test switch-on and first fitting. A similar analysis was performed using the preoperative audiometric thresholds as the input metric and the OR amplitude gradient groups as the output metric. The intention here was to investigate whether the gradient group could be influenced by floor effects. A patient with significant preoperative residual hearing can also lose a great deal of his or her residual hearing during surgery, while a patient with little preoperative residual hearing cannot lose much before reaching the maximum stimulation level.

## Results

### Hearing Threshold as Measured by Pure-Tone Audiometry

The average air conduction (AC) hearing threshold of patients at the low frequencies (250, 500, and 1000 Hz) before surgery was 52.2 dB HL ± 23.97 dB HL at 250 Hz, 65.3 dB HL ± 21.6 dB HL at 500 Hz, and 81.5 dB HL ± 16.8 dB HL at 1000 Hz. The pure-tone average for all three frequencies (PTA low) was 66.3 dB HL ± 17.7 dB HL. Individual PTA lows are given in Table 1.

Postoperatively, tone audiometry was performed at test switch-on (1–3 days after surgery) in a total of 112 of the 121 patient ears. For medical reasons (postoperative dizziness, tiredness, refusal by the patient), no audiometric threshold was determined in nine ears. The AC hearing threshold in the pure-tone audiogram during the postoperative test tone was, on average, 79.15 dB HL ± 21.7 dB HL (± standard deviation) at 250 Hz, 98.2 dB HL ± 19.9 dB HL at 500 Hz, and 109.7 dB HL ± 14.1 dB HL at 1000 Hz. The mean value over all three frequencies was 96.1 dB HL ± 16.6 dB HL. Evaluation of tone audiometry at first fitting (5 weeks after surgery) proved possible in a total of 100 patient ears. The remaining 21 patients underwent fitting in another clinical center, for example, or refused audiometry. The average AC hearing threshold at this time was 76.5 dB HL ± 26.2 dB HL at 250 Hz, 92.9 dB HL ± 23.4 dB HL at 500 Hz, and 100.97 dB HL ± 18.2 dB HL at 1000 Hz. The mean value over all three frequencies was 90.4 dB HL ± 20.4 HL, and the individual PTA lows are given in Table 1.

To allocate patients to hearing preservation groups, the difference between the low-tone hearing threshold (as measured by pure-tone audiometry) was calculated preoperatively, at test switch-on and at first fitting, and classified along the lines of Suhling et al. (2016). Patients’ distribution across the individual hearing preservation groups is provided in Table 3.

**Table 2.** Details of Stimulation and of Recording Parameters According to Our Protocol.

Frequency (Hz)	250	500	1000
Rise time (ms)	4	2	1
Plateau time (ms)	4	4	4
Fall time (ms)	4	2	1
Maximum stimulation level (dB nHL)	95	99	105
Lower filter (Hz)	100	200	300
Upper filter (Hz)	2000	2000	3000
Polarity	Rarefaction	Rarefaction	Rarefaction
Stimulation rate	19.1 Hz	19.1 Hz	19.1 Hz
Number of averaged trials per trace	10	10	10

**Table 3.** Distribution of Hearing Preservation Groups, Along the Lines of Suhling et al. (2016).

	Total TSO	TSO ΔPTA_low_ ≤ 15 dB HL	TSO 15 dB HL < ΔPTA_low_ ≤ 30 dB HL	TSO ΔPTA_low _> 30 dB HL	Total FF	FF ΔPTA_low_ ≤ 15 dB HL	FF 15 dB HL < ΔPTA_low_ ≤ 30 dB HL	FF ΔPTA_low _> 30 dB HL
Number	112	14 (12.5%)	45 (40.2%)	53 (47.3%)	100	32 (32%)	33 (33%)	35 (35%)

*Note:* The low-frequency PTA was calculated as the mean of the threshold at 250, 500, and 1000 Hz. TSO: test switch-on; FF: first fitting.

Differences in hearing threshold are given in the first three rows of Table 4. The differences between preoperative measurement and that at test switch-on were found to be significant at all three frequencies in the Wilcoxon analysis (250–1000 Hz: *Z* = −8.32; *p* < .001; *Z* = −8.21; *p* < .001; *Z* = −7.67; *p* < .001). Analysis using Spearman's correlation showed a strong positive correlation at all three frequencies, which also proved highly significant. Additionally, the differences between preoperative measurement and first fitting proved significant in the Wilcoxon analysis (*Z* = −8.998; *p* < .001; *Z* = 9.1; *p* < .001; *Z* = 9.02; *p* < .001). Spearman's correlation analysis revealed a highly significant positive correlation here as well, but it was slightly lower than at the test switch-on. Between test switch-on and first fitting, there was an average improvement in the threshold across all three frequencies, which also proved significant in the Wilcoxon analysis (*Z* = −0.87, *p* = .38 at 250 Hz; *Z* = −2.14, *p* = .03 at 500 Hz; *Z* = −4.51, *p* < .001 at 1 kHz). Spearman's correlation analysis also indicated a highly significant relationship.

**Table 4.** Mean Value of the Hearing Threshold Differences ± Standard Deviation in dB HL (for Audiometric Data) Respectively dB nHL (for ECochG Data).

	250 Hz	500 Hz	1000 Hz
Pre-OP audio versus post-OP audio TSO	27.8** ± 15.1	33.7** ± 16.9	28.6** ± 13.9
	*r* = .79**	*r* = .63**	*r* = .55**
Pre-OP audio versus post-OP audio FF	27.2** ± 19.9	30.4** ± 21.7	21.3** ± 16.7
	*r* = .64**	*r* = .47**	*r* = .42**
Post-OP audio TSO versus post-OP audio FF	−0.63** ± 18.8	−3.3** ± 19.3	−7.2* ± 19.7
	*r* = .69**	*r* = .58**	*r* = .395**
Pre-OP audio versus OR pre-ins	30.0* ± 27.3	18.1* ± 25.3	5.1* ± 24.6
	*r* = .06	*r* = .13	*r* = .04
OR pre-ins versus post-ins	3.0* ± 11.6	2.1* ± 11.2	1.8 ± 12.9
	*r* = .7**	*r* = .73 **	*r* = .81**
Pre-OP audio versus OR post-ins	33.3** ± 28.4	20.8** ± 25.6	6.7** ± 22.4
	*r* = .06	*r* = .11	*r* = .05
Post-OP audio TSO versus OR post-ins	8.1** ± 26.2	−10.7** ± 25.3	−20.8** ± 21.2
	*r* = .02	*r* = .01	*r* = .08
Post-OP audio FF versus OR post-ins	9.1* ± 27.4	−5.6* ± 24.1	−11.8** ± 22.94
	*r* = .15	*r* = .25*	*r* = .07

*Note: r*: correlation. Significance for the hearing threshold difference was calculated with Wilcoxon's analysis and is given in the respective top line, significance for *r* was calculated with Spearman's correlation analysis and is given in the respective bottom line.

*p < .05 (significant), **p < .01 (highly significant).

### Stimulus-Response Thresholds as Measured by ECochG

It proved possible to evaluate the measurement of stimulus response thresholds before and after insertion for a total of 107 patient ears. In 12 ears, pre-insertion recording yielded no response, so the remainder of the protocol was omitted. In the remaining two cases, post-insertion measurement was omitted for organizational reasons. The individual data are given in Table 1.

Electrocochleography measurements before insertion revealed a mean OR threshold at 250 Hz of 84.1 dB nHL ± 16.3 dB nHL, at 500 Hz of 84.8 dB nHL ± 16.1 dB nHL, and at 1000 Hz of 87.3 dB nHL ± 20.9 dB nHL. After insertion, the mean OR threshold was 86.7 dB nHL ± 16.6 dB nHL at 250 Hz, 86.5 dB nHL ± 15.6 dB nHL at 500 Hz, and 87.8 dB nHL ± 16.5 dB nHL at 1000 Hz. The mean OR threshold across all three frequencies was 85.7 dB nHL ± 15.1 dB nHL before insertion and 87.5 dB nHL ± 14.5 dB nHL after insertion. Accordingly, there was an average threshold shift of 2.2 dB nHL ± 8.2 dB nHL. This increase in the threshold of measurements before and after insertion was shown to be significant by the Wilcoxon test: *Z* = −2.43, *p* = .015.

Differences in both electrocochleographically determined thresholds before and after insertion exhibited, on average, only a minimal increase at all three frequencies (see Table 4). In the Wilcoxon test, these differences proved significant at the lower frequencies (250 Hz and 500 Hz; *Z* = −2.11; *p* = .04; *Z* = −2.27; *p* = .02), but not for the 1 kHz thresholds (*Z* = −1.27 *p* = .21). Spearman's correlation analysis confirmed a strong positive relationship which was highly significant (see Table 4). The difference between the mean OR threshold before and the mean OR threshold afterward also showed a minimal increase, which was proved significant (*Z* = −2.43; *p* = .02) by the Wilcoxon test. Individual differences between the mean OR thresholds at all three frequencies are given in Table 1.

### Relation Between Pure-Tone Audiometry and ECochG

**Figure 2.**
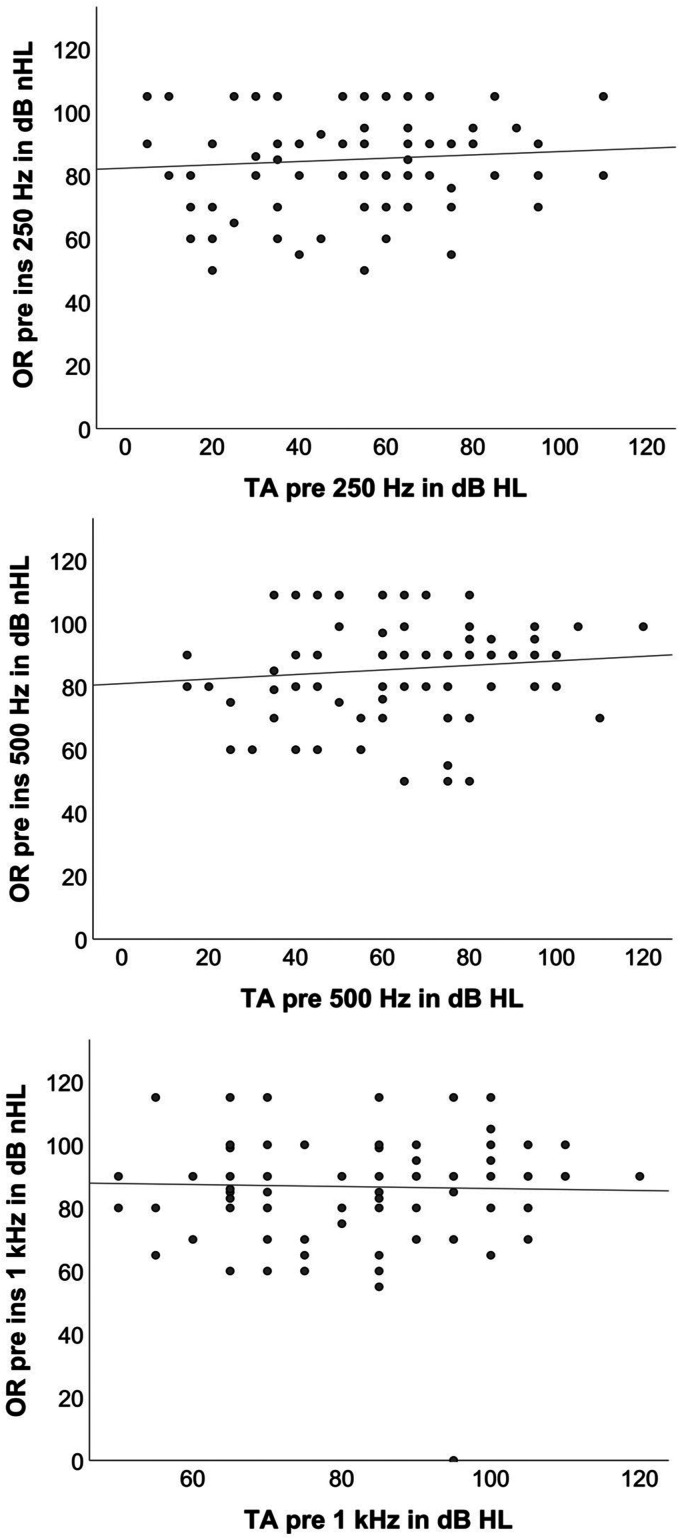
Scatter plots of threshold preoperatively at 250 Hz (top), 500 Hz (middle), and 1000 Hz (bottom) as obtained by tone audiometry (TA pre) and before insertion using ECochG (OR pre).

As a next step, correlations were investigated between the hearing thresholds measured by pure-tone audiometry preoperatively and the stimulus response thresholds determined by electrocochleography before insertion. A graphical analysis with scatterplots initially suggested a possible weak connection (see Figure 2). However, Spearman's correlation analysis revealed only a minimal positive correlation at all three frequencies (*r* = .058; *r* = .133; *r* = .037), which turned out to be not statistically significant (*p* = .58; *p* = .19; *p* = .72) (see Table 4).

**Figure 3.**
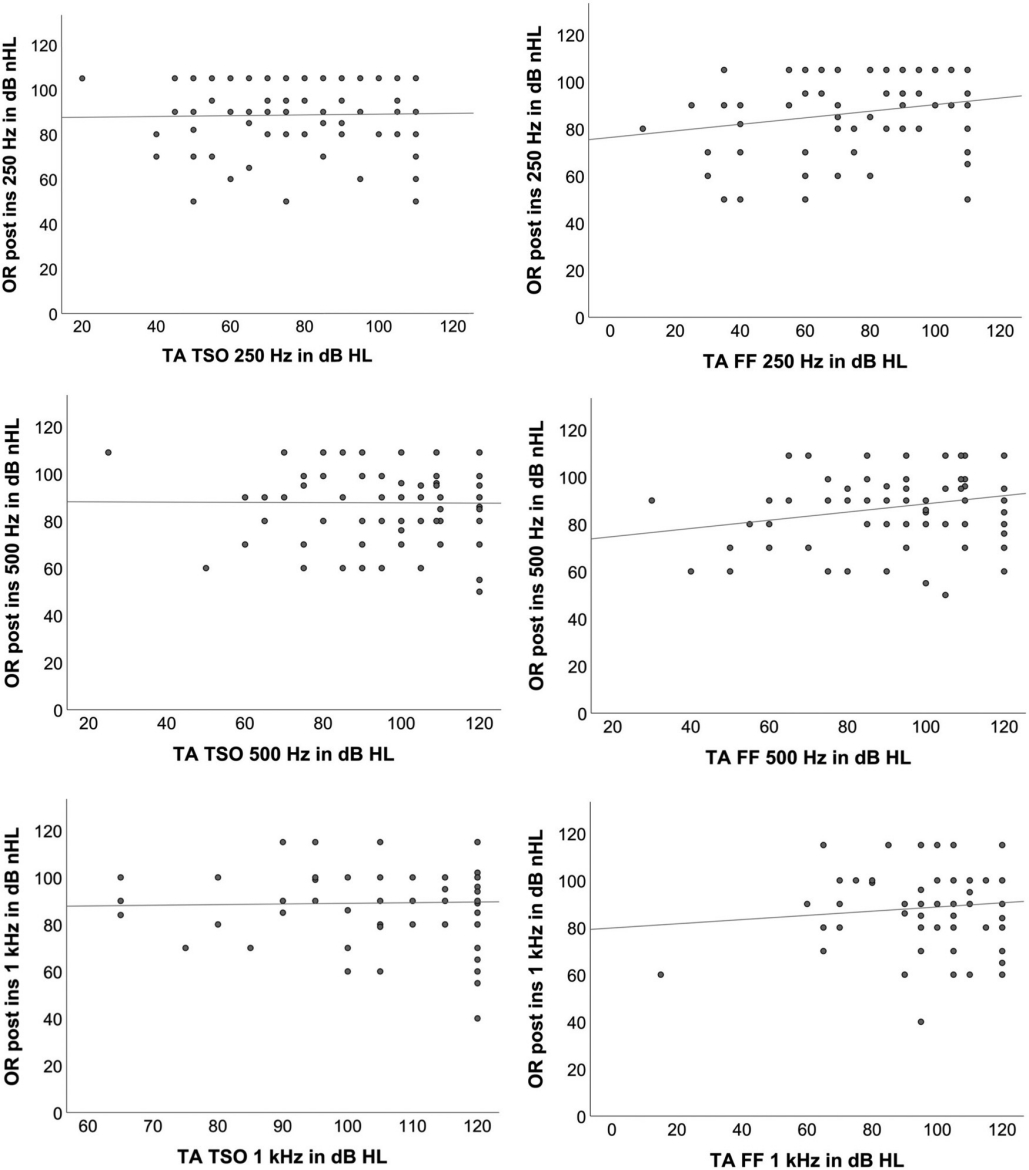
Scatter plots of thresholds postoperatively at 250 Hz, 500 Hz, and 1000 Hz obtained by tone audiometry (TA TSO, left panels) and after insertion using ECochG (OR post) and obtained by tone audiometry (TA FF, right panels) and after insertion using ECochG (OR post).

Next, the thresholds were compared electrocochleographically after insertion and, by audiometric means, postoperatively at the test switch-on. The graphical analysis in the scatter diagram (Figure 3) initially did not suggest any correlation between the values. This was also confirmed in Spearman's correlation analysis, which was only slightly positive and not significant (see Table 4). A closer examination of the differences in the thresholds showed that the OR threshold at 250 Hz was higher than the TA, whereas it was lower at 500 Hz and 1000 Hz. These differences were significant (*Z* = −2.68; *p* = .007; *Z* = −4.096; *p* < .001; *Z* = −9.76 *p* < .001). Next, the hearing thresholds as measured by pure-tone audiometry at first fitting, and the OR thresholds after insertion, were compared (see Figure 3). The results were similar to those of the previous analysis. The OR threshold at 250 Hz was higher than the TA, whereas it was lower at 500 Hz and 1000 Hz (see Table 4). These differences also proved significant across all three frequencies (*Z* = −2.42; *p* = .02; *Z* = −2.16; *p* = .03; *Z* = −4.07; *p* < .001).

Finally, shifts in hearing threshold between pre- and post-operative measurements were compared to shifts in OR threshold before and after insertion.

Pre- and post-operative differences in hearing threshold showed a threshold shift at all three frequencies, and also in the mean over all three frequencies (250 Hz: 27.2 dB ± 14.7 dB; 500 Hz: 32.9 dB ± 17.1 dB; 1000 Hz: 28.1 dB ± 14.2 dB; mean: 29.8 dB ± 13.0 dB). The Wilcoxon test revealed these to be significant for 250 Hz (*Z* = −8.99, *p* < .001), 500 Hz (*Z* = −9.1, *p* < .001) and 1000 Hz (*Z* = −9.02, *p* < .001), and also that the mean shift was significant (*Z* = −9.15, *p* ˂ .001).

Analysis of the OR threshold before and after insertion indicated a slightly increased threshold at all three frequencies (250 Hz: 2.8 dB ± 11.2 dB, 500 Hz: 1.9 dB ± 11.8 dB, 1 kHz: 1.8 dB ± 13.8 dB). The Wilcoxon test showed this to be significant for 250 Hz (*Z* = −2.31, *p* = .035) and 500 Hz (*Z* = −2.28, *p* = .023), but not for 1000 Hz (*Z* = −1.27, *p* = .206).

In addition, over all three frequencies the mean shifted slightly, with Wilcoxon testing again revealing that this shift (2.21 dB ± 8.16) was significant (*Z* = −2.43, *p* = .02).

Spearman's correlation analysis, as applied to the shift in OR threshold and the TA shift before surgery and at test switch-on, revealed only minimal correlations at all three frequencies (*r* = .02; *r* = −.02; *r* = .05), which turned out to be not statistically significant (*p* = .88; *p* = .83; *p* = .62).

Additionally, Spearman's analysis to detect any correlation between the shift in OR threshold and the TA shift before surgery and at first fitting, indicated also only a minimal correlation at all three frequencies (*r* = .18; *r* = .09; *r* = −.003), which proved not to be statistically significant (*p* = .13; *p* = .44; *p* = .98).

**Figure 4.**
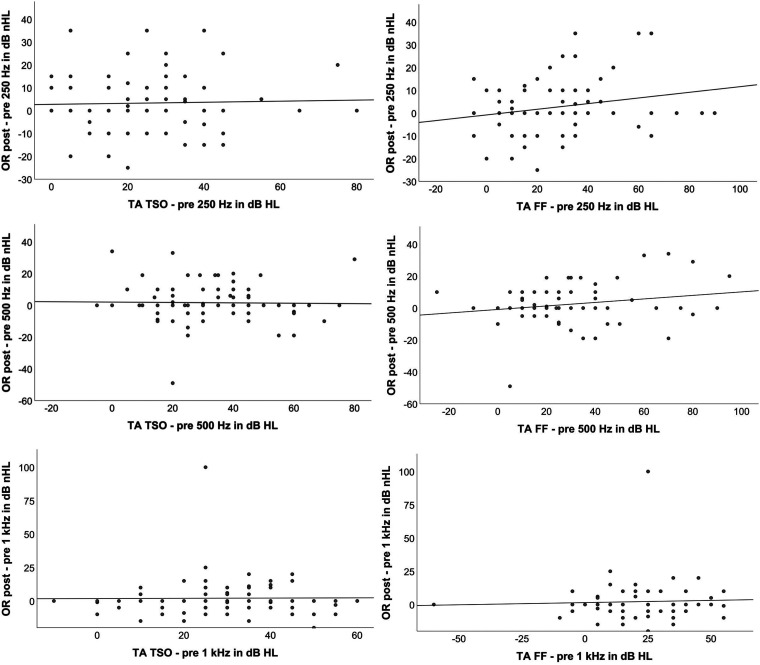
Scatter plots of threshold shifts postoperatively at 250 Hz, 500 Hz, and 1000 Hz obtained by tone audiometry (TA TSO, left panels and TA FF, right panels) and threshold shifts obtained using ECochG (OR post–pre) at 250 Hz, 500 Hz, and 1000 Hz.

The graphical analysis in the scatter diagrams (see Figure 4) initially did not suggest any correlation between the values. This was also confirmed in Spearman's correlation analysis which, at all three frequencies, was only slightly positive and not significant.

### Multivariate Analyses of the Relationships Between Preoperative Hearing Thresholds, ECochG Data, and Hearing Preservation Group

Table 5 provides details of the analysis concerning the hearing preservation group at test switch-on (TSO). The following significant differences were detected. Postoperative allocation to hearing preservation (HP) group 1 (0–15 dB) or 3 (> 30 dB) differed significantly depending on the preoperative hearing threshold at 250 Hz. Postoperative allocation to HP group 1 or 3, as well as HP group 2 (15–30 dB) or 3, showed a significant difference depending on the preoperative hearing threshold at 500 Hz. And postoperative allocation to HP group 1 or 2 differed significantly dependent on the pre-insertion OR threshold at 1000 Hz. Finally, the postoperative allocation to HP group 2 or 3 exhibited a significant difference depending on the mean audiometric threshold (PTA low) before surgery.

**Table 5.** Mutlivariate Analysis for Predicting the Hearing Preservation Group at TSO.

	Input metric	Levene test result, ***p***	Homogeneity of variance (***p*** > .05)	ANOVA type	Result, ***p***	Post hoc test	HP group	Result, ***p***	Significant difference detected (***p*** < .05)
**250 Hz**	Audiometric threshold	.232	Yes	Standard	.025	Tukey-HSD	1–2	.280	No
							2–3	.247	No
							1–3	.025	Yes
	OR threshold pre-ins	.348	Yes	Standard	.581	–	–	–	No
	OR threshold post-ins	.196	Yes	Standard	.700	–	–	–	No
	OR threshold diff.	.272	Yes	Standard	.725	–	–	–	No
**500 Hz**	Audiometric threshold	.049	No	Welch	.002	Games-Howell	1–2	.563	No
							2–3	.005	Yes
							1–3	.036	Yes
	OR threshold pre-ins	.185	Yes	Standard	.397	–	–	–	No
	OR threshold post-ins	.965	Yes	Standard	.787	–	–	–	No
	OR threshold diff.	.633	Yes	Standard	.594	–	–	–	No
**1** **000 Hz**	Audiometric threshold	.013	No	Welch	.064	–	–	–	No
	OR threshold pre-ins	.111	Yes	Standard	.047	Tukey-HSD	1–2	.036	Yes
							2–3	.097	No
							1–3	.122	No
	OR threshold post-ins	.232	Yes	Standard	.327	–	–	–	No
	OR threshold diff.	.004	No	Welch	.758	–	–	–	No
**Mean low freq**	Audiometric threshold	.002	No	Welch	.005	Games-Howell	1–2	.523	No
							2–3	.014	Yes
							1–3	.052	No
	OR threshold pre-ins	.288	Yes	Standard	.260	–	–	–	No
	OR threshold post-ins	.254	Yes	Standard	.679	–	–	–	No
	OR threshold diff.	.687	Yes	Standard	.806	–	–	–	No

In Table 6, details of the analysis regarding the hearing preservation group at first fitting (FF) are given. The following significant differences were found. The postoperative allocation to HP group 2 or 3 differed significantly depending on the OR threshold difference at 250 Hz. Postoperative allocation to HP group 1 or 3 showed a significant difference depending on the preoperative hearing threshold at 500 Hz. Postoperative allocation to HP group 1 or 3 differed significantly depending on the mean audiometric threshold (PTA low) before surgery.

**Table 6.** Mutlivariate Analysis for Predicting the Hearing Preservation Group at FF.

	Input metric	Levene test result, ***p***	Homogeneity of variance (***p*** > .05)	ANOVA type	Result, ***p***	Post hoc test	HP group	Result, ***p***	Significant difference detected (***p*** < .05)
250 Hz	Audiometric threshold	.986	Yes	Standard	.145	–	–	–	No
	OR threshold pre-ins	.648	Yes	Standard	.840	–	–	–	No
	OR threshold post-ins	.540	Yes	Standard	.072	–	–	–	No
	OR threshold diff.	.076	Yes	Standard	.025	Tukey's HSD	1–2	.789	No
							2–3	.028	Yes
							1–3	.114	No
500 Hz	Audiometric threshold	.480	Yes	Standard	.021	Tukey's HSD	1–2	.647	No
							2–3	.153	No
							1–3	.010	Yes
	OR threshold pre-ins	.959	Yes	Standard	.978	–	–	–	No
	OR threshold post-ins	.964	Yes	Standard	.231	–	–	–	No
	OR threshold diff.	.051	Yes	Standard	.594	–	–	–	No
1000 Hz	Audiometric threshold	.143	Yes	Standard	.079	–	–	–	No
	OR threshold pre-ins	.337	Yes	Standard	.191	–	–	–	No
	OR threshold post-ins	.598	Yes	Standard	.210	–	–	–	No
	OR threshold diff.	.441	Yes	Standard	.752	–	–	–	No
Mean low freq	Audiometric threshold	.232	Yes	Standard	.023	Tukey-HSD	1–2	.716	No
							2–3	.135	No
							1–3	.021	Yes
	OR threshold pre-ins	.493	Yes	Standard	.643	–	–	–	No
	OR threshold post-ins	.930	Yes	Standard	.134	–	–	–	No
	OR threshold diff.	.027	No	Welch	.060	–	–	–	No

### Behavior of Amplitudes During Insertion

In a subset of 70 patient ears out of a total of 121, an analysis of stimulus responses during insertion of the electrode proved possible. The remaining 51 ears either underwent intracochlear measurement or had incomplete documentation of measurement times, so that no regression could be formed. Due to the lack of comparability of data from intracochlear measurements, these were not considered further in this study. Another seven patients did not complete postoperative audiometry; this was for different reasons (missing the appointment, refusal to undergo measurement, etc.).

On average, the slope of the regression line had a value of 0.07 µV ± 0.33 µV; thus, according to the above definition, the average for all patient ears was shown to be a constant gradient. Division into four different amplitude gradient types resulted in the following distribution: 31 patient ears showed amplitude increasing in gradient during insertion (44.3%); in 15 ears, the gradient of the amplitude fell (21.4%); and, in 24 ears, there was a constant gradient (34.3%). In none of the patient's ears, a drop in amplitude with complete loss of signal occurred. A multivariate analysis of the relationship between preoperative audiometric thresholds and amplitude gradient was performed using a one-way ANOVA. Details are given in Table 7. Significant relationships were detected for differentiation between rising and constant amplitude gradient patterns with respect to preoperative audiometric thresholds at 250 Hz and at 500 Hz, as well as the mean low-frequency hearing (PTA low).

**Table 7.** Multivariate Analysis of the Preoperative Audiometric Hearing Threshold and the Amplitude Gradient Group.

Input metric	Levene test result, ***p***	Homogeneity of variance (***p*** > .05)	ANOVA type	Result, ***p***	Post hoc test	Amplitude gradient group	Result, ***p***	Significant difference detected (***p*** < .05)
Audiometric threshold at 250 Hz	.932	Yes	Standard	.028	Tukey's HSD	f – c	.113	No
						c – r	.033	Yes
						f – r	1.000	No
Audiometric threshold at 500 Hz	.612	Yes	Standard	.021	Tukey's HSD	f – c	.249	No
						c – r	.016	Yes
						f – r	.773	No
Audiometric threshold at 1000 Hz	.238	Yes	Standard	.392	–	–	–	No
Audiometric threshold PTA low	.912	Yes	Standard	.021	Tukey's HSD	f – c	.150	No
						c – r	.02	Yes
						f – r	.944	No

*Note:* f: falling; c: constant; r: rising.

**Figure 5.**
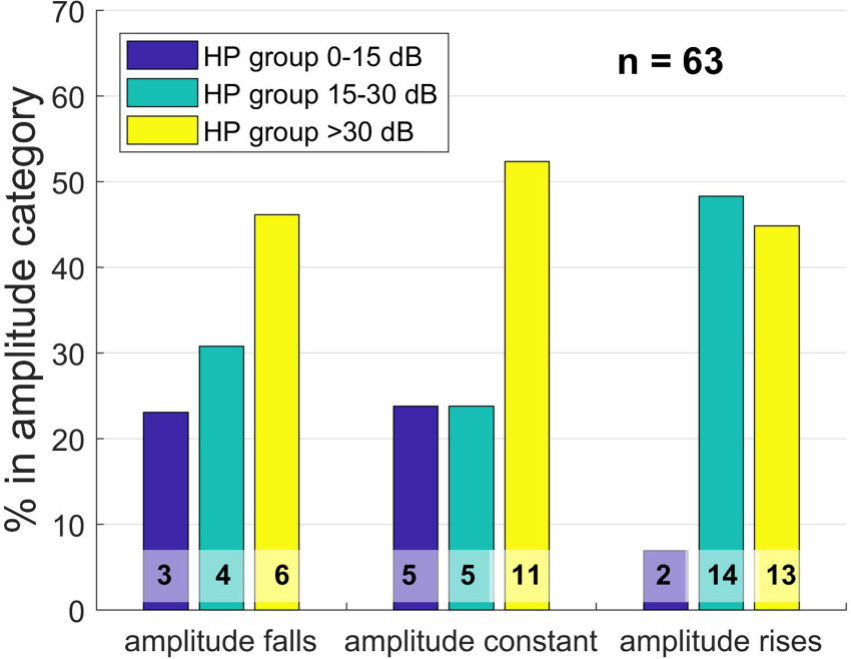
Distribution of hearing preservation (HP) groups by amplitude category at the time of test switch-on (TSO), 1–3 days after surgery, displayed as a bar plot. The HP group refers to the shift in low-frequency hearing threshold (PTA low) as a result of surgery. Group 0–15 dB means that the PTA low is a maximum of 15 dB worse postoperatively than preoperatively. In group 15–30 dB, the PTA low has deteriorated between 15 and 30 dB, and in group 3, the PTA low has deteriorated more than 30 dB. The Y axis gives the percentages of the data sets with respect to the size of the amplitude groups; the numbers at the bottom of the bars are absolute numbers.

The amplitude gradient types are shown in Figure 5, grouped by hearing preservation category at the time of postoperative test switch-on (TSO):

No significant correlation between amplitude behavior and hearing preservation group at the time of the test tone could be demonstrated in Pearson's chi-square test (X2 (4, *N* = 63) = 5.029; *p* = .284). In the Spearman correlation, there was a minimal negative correlation that was not statistically significant (*r*(63) = −.027; *p* = .837).

**Figure 6.**
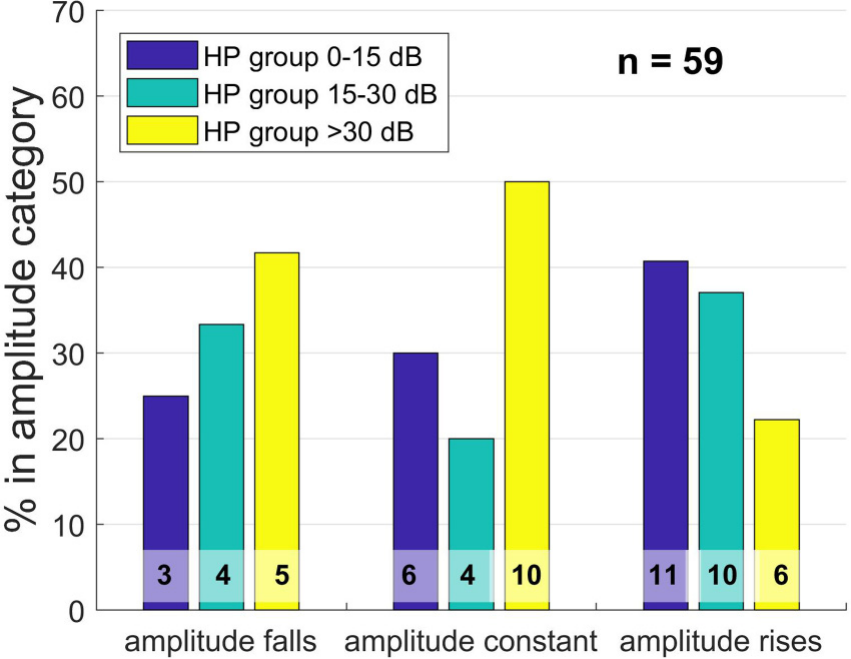
Distribution of hearing preservation (HP) groups by amplitude category at the time of first fitting (FF), 5 weeks after surgery, given as a bar plot. The HP group refers to the shift in low-frequency hearing threshold (PTA low) as a result of surgery. Group 0–15 dB means that the PTA low is a maximum of 15 dB worse postoperatively than preoperatively. In group 15–30 dB, the PTA low has deteriorated between 15 and 30 dB, and in group 3, the PTA low has deteriorated more than 30 dB. The Y axis gives the percentages of the data sets with respect to the size of the amplitude groups; the numbers at the bottom of the bars are absolute numbers.

Figure 6 shows the amplitude gradient types grouped by hearing preservation category at first fitting (FF), which typically took place 5 weeks after the surgery:

No significant link between amplitude behavior and hearing preservation group at first fitting could be demonstrated in the Pearson chi-square test (X2 (4, *N* = 59) = 4.522; *p* = .34). Spearman's test showed a weak positive correlation that was not statistically significant (*r*(59) = .214; *p* = .104).

## Discussion

In this work, we retrospectively analyzed extracochlear, intraoperative ECochG recordings of ORs before, during, and after CI electrode insertion. This method was introduced in our department, and at other clinical centers, to monitor the status of the cochlea intraoperatively and thus to improve patients’ residual hearing preservation. One limitation of OR recording is that it only reflects the state of the cochlea, whereas the auditory pathway also includes other anatomical structures. Therefore, the preservation of OR signals does not necessarily have to go hand in hand with the preservation of residual hearing (Campbell, Kaicer, Briggs & O’Leary, 2015; Fitzpatrick et al., 2014; Haumann et al., 2019).

The aim of this analysis was to investigate correlations between extracochlear ECochG measurements and residual hearing preservation in a patient population that is large enough to give a statistically meaningful outcome. Many of the studies published to date show a mixed picture of smaller patient cohorts, and most studies investigate the ECochG amplitude or threshold. Compelling correlations have not yet been found with these metrics. Thus, in this analysis, we first evaluated how large the sample size needed to obtain statistically significant results, based on the data described in Haumann et al. (2019). This analysis indicated a required sample size of 112 ears, which we achieved with our 121 datasets.

A disadvantage of all intraoperative measurement methods is that, while they may represent the status of the cochlea correctly at the end of surgery, postoperative processes can also impair the patient's residual hearing ability. Therefore, a constant signal amplitude during electrode insertion, or preserved stimulus-response thresholds in conjunction with later deterioration in hearing, can mean that residual hearing is still present at the end of the operation and is subsequently damaged by postoperative processes, or that the measurement did not succeed as intended (Haumann et al., 2019). Usually, the first postoperative audiometric evaluation takes place 1–3 days after surgery. Both air conduction (AC) and bone conduction (BC) thresholds are already adversely affected by postoperative processes when recorded at this time. AC thresholds, in particular, may also be inaccurate due to temporary middle ear effusion. However, at the next routine clinical evaluation time point 5 weeks after surgery, the fluid in the middle ear has cleared, but at this stage, the thresholds are more influenced by electrode insertion trauma and a foreign-body response. This lack of a good measure for residual hearing immediately after surgery prevents us from investigating whether intraoperative ECochG measurements are correlated with electrode insertion trauma leading to residual hearing loss following cochlear implantation. In a close-to-immediate postoperative BC evaluation within 5 hours after surgery the detected correlations were higher, indicating that the method itself seems to work (Saoji et al., 2022). Also, one statement that has been included in publications several times is that a constant signal does not allow any conclusions to be drawn, while a distinct drop in signal indicates a relevant trauma, this being accompanied by a loss of residual hearing (Adunka et al., 2016; Dalbert et al., 2015; Radeloff et al., 2012). This could also be an indication that the method basically works.

Our data showed that, at test switch-on, residual low-frequency hearing of the patients’ ears corresponded in 12.5% of cases to hearing preservation group 1 (0–15 dB drop), in 40.2% of cases to group 2 (˃ 15–30 dB drop), and in 47.3% of cases to group 3 (> 30 dB drop). At first fitting, residual low-frequency hearing was allocated in 32% of cases to group 1, in 33% of cases to group 2, and in 35% of cases to group 3. Hence, the residual hearing measurements obtained for the patients’ ears were approximately within the range reported in the literature (Hunter et al., 2016; Lenarz et al., 2009; Suhling et al., 2016). In our retrospective analysis, all electrodes from all CI manufacturers were included, so that individual group sizes are not sufficient for detailed analysis of residual hearing preservation on a per-electrode basis. Nevertheless, the focus of this work is on the ECochG method and its usefulness in preserving residual hearing, irrespective of the CI electrode used. The recorded OR thresholds before and after CI electrode insertion remain fairly stable for the whole group.

Analysis of the differences between pure-tone audiometry and OR thresholds shows the results to be generally similar to those reported in (Haumann et al., 2019). All OR thresholds before CI electrode insertion were worse than the AC thresholds before surgery. The differences were largest for 250 Hz and smallest for 1000 Hz; however, in the larger cohort investigated in the present work, the standard deviation was higher and thus the correlation was lower than in the small sample investigated by Haumann et al. (2019). The same tendencies were found for postoperative comparisons, but the postoperative AC thresholds deteriorated more than the post-insertion OR thresholds. For 250 Hz, the OR thresholds directly after CI electrode insertion were still worse than the AC thresholds postoperatively at both TSO and first fitting. By contrast, OR thresholds for 500 Hz and 1000 Hz were better.

A key question concerns the extent to which subsequent residual hearing can be predicted with the aid of pre- and intra-operative variables, with this study primarily targeting intraoperative factors to improve residual hearing preservation during surgery. A few significant relationships were detected between preoperative audiometric thresholds at 250 and 500 Hz and both the mean low-frequency threshold and the hearing preservation group at the test switch-on. Additionally, significant relationships were found between preoperative audiometric thresholds at 500 Hz and both the mean low-frequency threshold and the hearing preservation group at the first fitting. More relevant for the analysis of OR amplitudes is the significant relationship between pre-insertion OR threshold at 1000 Hz and allocation to hearing preservation group 1 (0–15 dB) and 2 (15–30 dB), but this relationship was no longer evident by the time of first fitting.

Of particular interest for the purpose of monitoring cochlear function is the relationship between the measured threshold shifts. If the OR threshold is the same after insertion as before, the postoperative pure-tone audiogram should ideally be the same as the preoperative one. If, however, the OR threshold has changed after electrode insertion, it would be desirable to draw clear conclusions about the change in the pure-tone threshold. As previously described, postoperative processes also have a significant influence and make data interpretation more difficult. Nevertheless, in the case of a shift in the OR thresholds, one would expect a correlation with the later shift as measured by pure-tone audiometry. This was partially evident in our data, with a significant relationship detectable between OR threshold shift at 250 Hz and allocation to HP group 2 (15–30 dB) or 3 (> 30 dB) at first fitting. For the other two frequencies (500 and 1000 Hz), no significant relationship could be detected. Significant two-variable correlations were not found, so a change in OR threshold—at least at 250 Hz—does not explain hearing loss as a stand-alone measurement, but appears to add some information about residual hearing loss, at least in terms of the differentiation between medium hearing preservation and complete loss. The fact that the differences are significant at 250 Hz, and not at 500 or 1000 Hz, could possibly be related to the fact that many CI candidates hear best at 250 Hz, whereas at 500 and 1000 Hz there may be an incipient floor effect regarding the upper measurement limit, which could falsify the analysis. Another possibility could be that those hair cells in the inner ear which are most sensitive to 250 Hz are located so deep in the cochlea that almost no electrode array reaches or exceeds them during insertion (Buechner et al., 2022; Haumann, Timm, Büchner, Lenarz & Salcher, 2024). Effects that reduce stimulus responses at 250 Hz should, therefore, genuinely be related to cochlear health and not to “blockages” of the basilar membrane caused by mechanical damping from the inserted electrode.

Next, the behavior of the OR amplitude during CI electrode insertion was analyzed. Here, the amplitude curve was classified into different behavior groups and examined as to the extent of any relationship with the residual hearing preservation group. Certain features become apparent when examining the gradients. With regard to the HP category at TSO, a falling amplitude was associated with fewer ears being allocated to the best HP group, more ears being allocated to the medium HP group, and most patients being allocated to the worst group; this is in line with expectations. Where the amplitude was constant, the same numbers of ears were allocated to the medium and best HP group, but more than half of the ears were allocated to the worst HP group. This corroborated previous findings that a constant amplitude provides little information about residual hearing preservation. Furthermore, many ears of all amplitude gradient groups were allocated to the worst HP group at the time of TSO, indicating that fluid in the middle ear is probably of significance. With increasing amplitude, only very few ears were allocated to the best HP group, which was somewhat surprising since one might expect increasing activity in the inner ear being associated with good residual hearing preservation rather than considerable deterioration. Nevertheless, the analysis showed that the differentiation between rising and constant gradient patterns was significantly associated with preoperative residual hearing at the very low frequencies, whereby ears with worse hearing tending toward rising amplitudes and those with better hearing tending toward constant amplitudes. Thus, a bias effect could also be involved. If a patient starts with significant residual hearing, he or she may lose more hearing, or the effect of fluid in the middle ear may be stronger than when there is little residual hearing before surgery.

With regard to HP group at first fitting, the results are much more consistent, even if statistically significant correlations could not be detected. The percentage of hearing preserved cases in the “amplitude falls” category is lowest, the “amplitude constant” category has more such cases, and the “amplitude rises” category has the highest percentage of hearing preserved cases. Conversely, the proportion of worst HP cases is—at least—lowest in the “amplitude rises” category, whereas that of medium HP cases is lowest in the “amplitude constant” group. Moreover, this supports the finding that a constant amplitude gradient does not allow confident conclusions to be drawn, but that a rising gradient would seem to favor hearing preservation and that a falling course appears to be associated with comprehensive hearing loss.

Extracochlear ECochG involves keeping the recording electrode in place during insertion of the CI electrode, so the expected optimum behavior would theoretically be constancy of amplitude, with the insertion of the CI electrode not affecting the state of the cochlea and hence subsequently not impacting residual hearing. This is not confirmed by our data, which is in line with the findings of other research groups (Dalbert et al., 2015; Radeloff et al., 2012). However, neither does deterioration of the OR amplitude necessarily lead to poor hearing preservation, although our data confirms that in this instance the risk is higher. In our cases, no complete loss of the signals occurred. Corresponding to complete loss, we would expect a severe decline in hearing but, as this did not occur, we were of course not able to demonstrate this based on our data. One interesting pattern is the increase in amplitude. A reason for this could be that the closed round window, in particular, dampens the vibrations in the inner ear, so that the signals can be measured best when the round window is open. Another possible explanation is that opening the round window may lead to leakage of some perilymph, which would technically change the recording process from extracochlear to intracochlear in nature, yielding larger response amplitudes (Giardina et al., 2018; Walia et al., 2022). A further potential explanation for an increase in amplitude during insertion is that the cochlea of the individual patient reacts very sensitively to changes so that the electrode insertion initially results in a strong stimulus response but the cochlear structures incur greater damage. However, our data did indicate that the cochleae of these patients regenerate to an extent after a little more time. Dalbert et al. (2015, 2018) also report that in some patients the OR amplitude rises during insertion, but they do not comment on the impact in terms of these subjects’ hearing preservation.

Another research group has investigated the relationship between phase changes and hearing preservation, with a phase shift of 90° or more being associated with a significantly larger deterioration of the residual hearing (Suntinger et al., 2022). Here, ECochG recordings were performed before and after CI electrode insertion. One trace consisted of 400 averaged trials, presented at a stimulation rate of 23.3 Hz, yielding a measurement time of 17 s per trace. The more averaged trials a measurement has, the cleaner the measured signal is; however, a longer measurement duration has an adverse effect on real-time measurement during insertion. Accordingly, in Suntinger et al.'s study, it was measured before and after insertion, whereas in our set-up we placed more emphasis on real-time feedback during insertion. For this reason, we measured with only 10 averaged trials per trace, which resulted in a measurement time of 0.5 s per trace at our stimulation frequency of 19.1 Hz. This is sufficient for visual evaluation of the signals, with an immediate estimate of the amplitude change for the surgeon. However, this approach has weaknesses in terms of a thorough offline analysis, so unfortunately, we cannot use our data to test this interesting approach by the above research group. For future measurements, a scenario would be conceivable where measurements are taken with a large number of averaged trials per trace before and after insertion to clearly detect phase shifts, and with significantly less averaged trials being used during insertion to obtain real-time feedback. Here, a compromise would have to be made between clean phase detection and short measurement time.

## Conclusion

In this retrospective analysis, the suitability of intraoperative extracochlear ECochG measurements for monitoring cochlear function and, ultimately, residual hearing preservation during a hearing-preserving CI operation, was investigated on a large group of patients. The size of the population was calculated such that statistically reliable statements are possible.

With extracochlear ECochG, recordings were made of both the OR thresholds before and after CI electrode insertion and the behavior of the OR amplitude during CI electrode insertion. Extracochlear ECochG threshold changes before and after CI insertion were relatively small and did not independently correlate well with hearing preservation, but at the very low frequency of 250 Hz, this does add some information. Extracochlear ECochG amplitude changes throughout CI insertion, on average, were mostly stable and also relatively small. Some tendencies were detected, but no significant relationships were observed. Rising amplitudes seem to be favorable for better hearing preservation and falling amplitudes would appear to be disadvantageous, but constant amplitudes do not seem to allow for stringent predictions. Early postoperative hearing (1–3 days after surgery) is poorly associated with ECochG change and is probably not clinically useful in the context of ECochG prediction models of hearing preservation. Extracochlear ECochG may be a very specific (but not a sensitive) marker of ultimate hearing loss, as we demonstrated few to no ECochG changes intraoperatively but found a wide range of postoperative hearing outcomes.

Overall, the promise inherent in monitoring of residual hearing during CI surgery using extracochlear ECochG seems to be only partially confirmed. The key benefit of extracochlear recordings is that it can be performed before, during, and after surgery. Also, different frequency responses can be measured with an electrode that lies outside the structures where the responses are generated. This has the advantage that it is less invasive and, due to the distance from the generator, every minimal fluctuation may not be captured and hence the signal could be more stable. The main drawback is that the derivation is very wide, which reduces the effect of hearing loss. However, a preserved OR seems to indicate that residual hearing is still possible. Therefore, it should be considered whether the benefit is worth the effort or, perhaps, whether other methods offer larger benefits. Combinations of different measuring methods would also be conceivable to obtain the best possible benefit for the patient.
